# Characterising spontaneous bacterial peritonitis in liver cirrhosis and hepatocellular carcinoma in southwestern Nigeria

**DOI:** 10.4314/gmj.v59i2.2

**Published:** 2025-06

**Authors:** Abosede G Adeyeye, Adeyemi T Adeyemo, Victor O Adeyeye, Temitope O Ojo, Olusegun Adekanle, Anthony C Anuforo, Aaron O Aboderin, Dennis A Ndububa

**Affiliations:** 1 Gastroenterology and hepatology Unit, Department of Medicine, Osun State University Teaching Hospital, Osogbo, Nigeria; 2 Department of Medical Microbiology & Parasitology, Obafemi Awolowo University Teaching Hospital Complex, Ile-Ife, Nigeria; 3 Department of Medicine, Babcock University Teaching Hospital, Ilishan Remo, Nigeria; 4 Department of Community Health, Obafemi Awolowo University, Ile-Ife, Nigeria; 5 Gastroenterology and hepatology Unit, Department of Medicine, Obafemi Awolowo University Teaching Hospital Complex, Ile-Ife, Nigeria; 6 Department of Medical Microbiology & Parasitology, Obafemi Awolowo University, Ile-Ife, Nigeria

**Keywords:** Prevalence, spontaneous bacterial peritonitis, liver cirrhosis and HCC, Ile-Ife, Nigeria

## Abstract

**Objectives:**

This study determined the profile of spontaneous bacterial peritonitis (SBP) and its variants in patients with decompensated liver cirrhosis and hepatocellular carcinoma (HCC).

**Design:**

A descriptive cross-sectional study

**Setting:**

The study was conducted in a tertiary hospital.

**Participants:**

Patients with decompensated liver cirrhosis and HCC above 18 years.

**Interventions:**

Ascitic fluid (AF) samples were taken for cell count and culture using a BACTEC culture bottle. Sensitivity patterns and ascitic fluid total protein and albumin levels were also assessed.

**Main outcome measures:**

Clinical profile of SBP, organisms isolated from the ascitic fluid and sensitivity of isolated organisms to antibiotics.

**Results:**

One hundred and six (106) participants were recruited. Seventy (66%) had liver cirrhosis, and 36 (34%) had HCC. The mean age was 50.08±12.66 years. Eighty-nine (84%) were males and 17(16%) were females. The overall prevalence of SBP was 29.2% (n = 31). Classical SBP was 5(4.7%), CNNA 20(18.9%) and monobacterial ascites 6(5.7%). The Gram-positive isolates were *Staphylococcus aureus* 5(45.5%) – [2(18.2%) MRSA, 3(27.3%) MSSA] while the Gram-negative organisms were *E. coli* 3(27.3%), *Acinectobacter* 2(18.2%) and *Bulkholderia cepacia* 1 (9.1%). Gram-negative bacteria showed absolute resistance to cephalosporins but were all susceptible to meropenem. Gram-positive bacteria showed 100% susceptibility to linezolid, vancomycin and daptomycin. Gram-positive bacteria also showed low resistance to fluoroquinolones (20%). Multi-drug resistance pattern was reported for MRSA, *Acinetobacter baumannii* and *Bulkholderia cepacia*.

**Conclusion:**

SBP is a common complication in patients with decompensated CLD. Guided antibiotic treatment should be encouraged, particularly in light of the emergence of multidrug resistance patterns.

**Funding:**

None declared

## Introduction

Spontaneous bacterial peritonitis (SBP) is a common bacterial infection in patients with cirrhosis of the liver.^1^,^2^ It was first described by Conn and Fessel in 1971 as a syndrome of infected ascitic fluid in patients with hepatic cirrhosis.^3^ SBP accounts for 10-30% of all reported bacterial infections in hospitalised patients.^4^ The diagnosis of SBP is made when there is a positive ascitic fluid culture and an elevated ascitic fluid absolute polymorphonuclear neutrophil count (i.e., at least 250/mm^3^ [0.25 × 10^9^/L]) without evidence of an intra-abdominal surgically treatable source of infection.^5^,^6^

Patients with decompensated chronic liver disease (CLD) are vulnerable to bacterial infections due to defects in various host immune defence mechanisms.^7^ Bacteria most commonly isolated from ascitic fluid in patients with spontaneous bacterial peritonitis (SBP) are usually those of the normal intestinal flora.^8^,^9^ Seventy per cent of patients with cirrhosis who have SBP are in Child Pugh class C, which is the most important risk factor for SBP.^8^ The symptoms and signs of SBP are subtle compared with those of patients who have surgical peritonitis in the absence of ascites. SBP may be asymptomatic in approximately 10-32% of cases, particularly in outpatient settings.^5^,^9^ Symptoms and signs that may be present in patients with SBP include fever, abdominal pain, altered mental status, abdominal tenderness, diarrhoea, paralytic ileus, hypotension and hypothermia.^10^ Because of the significant risk of adverse outcomes related to SBP, identifying high-risk patients for early and appropriate treatment will help in reducing the morbidity and mortality associated with SBP. This study therefore determined the profile of spontaneous bacterial peritonitis (SBP) and its variants in patients with decompensated liver cirrhosis and hepatocellular carcinoma (HCC).

## Methods

### Study design

This was a hospital-based, cross-sectional study conducted over 18 months from September 2020 to March 2022. The study was conducted at the emergency ward, medical inpatient wards, medical outpatients' clinic, and Department of Medical Microbiology of the Obafemi Awolowo University Teaching Hospitals Complex (OAUTHC), Ile-Ife, Nigeria, after written informed consent was obtained from the patient.

### Study population

Patients with complaints such as abdominal swelling, abdominal pain with or without yellowness of the sclera, weight loss with examination findings of peripheral stigmata of chronic liver disease, abdominal examination findings of reduced liver span, ascites and ultrasonographic findings of shrunken liver with coarse parenchymal echotexture and wavy outline were recruited as liver cirrhosis cases while patients with abdominal examination findings of tender, hard, nodular hepatomegaly with or without hepatic bruit and with classical features of HCC on abdominal triphasic computed tomography and/or elevated alpha-fetoprotein having ascites were recruited as hepatocellular cancer cases. Excluded were patients with secondary bacterial peritonitis, ascites due to renal and cardiac causes, CLD other than liver cirrhosis and HCC and fulminant hepatic failure from any cause with ascites. A detailed history and clinical examination were conducted.

### Specimen collection and processing

Abdominal paracentesis was done under aseptic conditions. Ascitic fluid (AF) was physically observed for colour and turbidity. AF was then distributed as follows: 10 mL into a BACTEC aerobic bottle for culture, which was followed by bacterial identification and antibiotic sensitivity testing using standard microbiology laboratory protocols. Five millilitres of AF were collected into an Ethylene Diamine Tetra-Acetate (EDTA) bottle and analysed within six hours for white cell count using a manual counting method with a modified Neubauer chamber. 5mls into a lithium heparin bottle for total protein and albumin quantification. Blood samples were also collected for liver function tests, serum sodium and creatinine levels, prothrombin time (PT), INR, hepatitis B surface antigen, and hepatitis C virus antibodies. Radiological imaging, including ultrasonography and/or computed tomography, was also performed to confirm the cases.

### Definitions of SBP and its variants

For the purpose of the study, the following definitions of AF infection were used:

1. Classical spontaneous bacterial peritonitis: presence of AF neutrophil count of ≥ 250 cells/mm^3^ and positive AF culture for a single organism.
2. Culture-negative neutrocytic ascites (CNNA): presence of AF neutrophil count ≥ 250 cells/mm^3^ and negative AF culture.
3. Monomicrobial non-neutrocytic bacterascites: presence of AF neutrophil count < 250 cells/mm^3^ and positive AF culture for a single organism.

### Data analysis

Data analysis was conducted using SPSS version 25 software (IBM SPSS Statistics for Windows, Version 25.0; Armonk, NY: IBM Corp.). Prevalence of SBP was represented using percentages, and the frequency of the variants of SBP was presented as a frequency table and barcharts. The association between selected patient characteristics and the occurrence of spontaneous bacterial peritonitis was assessed using the chi-squared test. However, in instances where the expected count was less than 5 in more than 25% of the cells, a likelihood ratio (LR) test was conducted. Descriptive analysis was employed to present the laboratory culture and antibiotic sensitivity results. A probability of p<0.05 was considered statistically significant.

### Ethical approval

Approval from the Ethics and Research Committee of Obafemi Awolowo University Teaching Hospital was sought and obtained prior to the commencement of the study, with protocol number ERC/2020/07/06. Informed consent was obtained from participants.

## Results

One hundred and ten (110) study participants were recruited. Four (4) were excluded as the cause of ascites was not liver-related, and thus, 106 participants eventually completed the study. The age range of participants was 20-70 years, with a mean (SD) of 50.08±12.66. Participants in the 40-49 age group comprised 30 (28.3%) of the subjects, accounting for the highest proportion. There were more men than women (84.0% vs. 16.0%), with a male-to-female ratio of 5.25:1, and a majority of inpatients (70, 66.0%). Common symptoms among study participants were abdominal swelling, fatigue, and weight loss, reported in 104 (98.1%), 87 (82.1%), and 69 (65.1%) participants, respectively. Fever occurred in 28 (26.4%), jaundice in 67 (63.2%), haematemesis in 12 (11.3%), melaena in 20 (18.9%), renal impairment in 19 (17.9%), and features of hepatic encephalopathy in 26 (24.5%). Thirty-nine (36.8%) had a significant history of alcohol use.

Seventy (66.0%) of the study participants were hepatitis B positive, while one (0.9%) patient had hepatitis C infection. On abdominal ultrasonography, 61(57.5%) had enlarged liver, 17(16%) had shrunken liver and 28(26.4%) had normal liver span. Ultrasound also showed splenomegaly in 11 (10.4%), coarse parenchymal liver echogenicity in 97 (91.5%), and hepatic nodules in 33 (31.1%) study participants. About half (54; 50.9%) of the study participants were in Child-Turcotte-Pugh (CTP) class C, while 45(42.5%) were in class B and 7(6.6%) were in Class A. (Table 1)

**Table 1:** Demographics and clinical characteristics of the study participants (N=106)

Characteristics	Frequency (n(%))
**Age**	
20-29yrs	4(3.8)
30-39yrs	17(16.0)
40-49yrs	30(28.3)
50-59yrs	29(27.4)
60-69yrs	19(17.9)
70yrs and above	7(6.6)
**Sex**	
Male	89(84.0)
Female	17(16.0)
**Admission status**	
**Inpatient**	70(66.0)
**Outpatient**	36(34.0)
**Fever**	28(26.4)
**Abdominal pain**	56(52.8)
**Abdominal Swelling**	104(98.1)
**Fatigue**	87(82.1)
**Yellowness of the eyes**	67(63.2)
**Weight loss**	69(65.1)
**Hematemesis**	12(11.3)
**Passage of Melena**	20(18.9)
**Hepatic Encephalopathy**	26(24.5)
**Hepatitis B positive**	70(66.0)
**Hepatitis C positive**	1(0.9)
**Liver span**	
**Normal**	28(26.4)
**Shrunken**	17(16.0)
**Enlarged**	61(57.5)
**Splenomegaly**	11(10.4)
**Liver Echogenicity/Nodules**	
**Coarse**	97(91.5)
**Nodules**	33(31.1)
**CTP**	
**Class A**	7(6.6)
**Class B**	45(42.5)
**Class C**	54(50.9)
**Diagnosis**	
**Liver cirrhosis**	70(66.0)
**HCC**	36(34.0)

*CTP=Child-Turcotte-Pugh; HCC=Hepatocellular carcinoma

NB: Multiple responses allowed

The overall prevalence of SBP was 29.2%, with classical SBP accounting for 5 (4.7%), 20 (18.9%) had culture-negative neutrocytic ascites, while monomicrobial non-neutrocytic bacterascites accounted for 6 (5.7%). (Table 2)

**Table 2:** Frequency of the variants of SBP among patients with liver cirrhosis and HCC

SBP Status	Liver cirrhosis n=70(%)	HCC n=36(%)	Combined Liver cirrhosis and HCC n=106(%)
**Classical SBP**	4 (5.7)	1 (2.8)	5 (4.7)
**CNNA**	14 (20.0)	6 (16.7)	20 (18.9)
**MNB**	3 (4.3)	3 (8.3)	6 (5.7)

SBP=Spontaneous Bacterial Peritonitis; CNNA=Culture Negative Neutrocytic Ascites;

MNB=Mono-microbial non-neutrocytic Bacterascites

The major symptoms seen in the study participants with SBP were generalised body weakness, abdominal pain and fever. SBP accounted for 28.7%, 32.7% and 39.3% of these symptoms, respectively. There was no statistically significant difference in the clinical manifestations between patients with SBP and those without SBP. SBP was also seen in participants with symptoms like haematemesis 3 (25%), passage of melaena 5 (25%) and reduced urinary output 6 (31.6%), suggesting upper GI bleeding and acute kidney injury. (Table 3)

**Table 3:** Clinical presentation and drug use of participants with and without SBP

Variables	SBP N=31 (%)	NO SBP N=75 (%)	Statistical comparison
**Fever**			
**Yes**	11 (39.3)	17 (60.7)	χ^2^=1.853
**No**	20 (25.6)	58 (74.4)	p=0.173
**Abdominal pain**			
**Yes**	18 (32.7)	37 (67.3)	χ^2^=0.670
**No**	13 (25.5)	38 (74.5)	p=0.413
**Generalized body weakness**			
**Yes**	25 (28.7)	62 (71.3)	χ^2^=0.610
**No**	6 (31.6)	13 (68.4)	p=0.805
**Haematemesis**			
**Yes**	3 (25.0)	9 (75.0)	χ^2^=0.118
**No**	28 (29.8)	66 (70.2)	p=0.731
**Passage of Melaena**			
**Yes**	5 (25.0)	15 (75.0)	χ^2^=0.215
**No**	26 (30.2)	60 (69.8)	p=0.643
**Reduced Urinary Output**			
**Yes**	6 (31.6)	13 (68.4)	χ^2^=0.061
**No**	25 (28.7)	62 (71.3)	p=0.805
**Use of Beta blocker**			
**Yes**	0 (0.0)	2 (100.0)	LR=1.400
**No**	31 (29.8)	73(70.2)	p=0.237
**Antibiotic use**			
**Yes**	7 (24.1)	22 (75.9)	χ^2^=0.503
**No**	24 (31.2)	53 (68.8)	p=0.478

The majority of the study participants with SBP were in CTP Class C, 58.1% (18/31), and also had hypoalbuminaemia, 96.8% (30/31), and hyperbilirubinaemia, 80.6% (25/31). There was no statistically significant difference in risk factors among the clinical and laboratory factors studied in the two patient groups. (Table 4)

**Table 4:** Risk factors associated with SBP among study participants

VARIABLES		SBP N=31(%)	NO SBP N=75(%)	Statistical comparison
**Bilirubin**	**<20**	6(20.0)	24(80.0)	χ^2^=1.728
**(umol/L)**	**>20**	25(32.9)	51(67.1)	P=0.189
**Albumin**	**<35**	30 (32.6)	62(67.4)	χ^2^=3.803
**(g/L)**	**35-50**	1(7.1)	13(92.9)	P=0.051
**INR**	**<0.9**	0(0)	1(100.0)	χ^2^=2.141
	**0.9-1.2**	8(21.6)	29(78.4)	P=0.343
	**>1.2**	23(33.8)	45(66.2)	
**Platelet count**	**<90,000**	7(46.7)	8(53.3)	LR=2.409
**(per cmm^3^)**	**90,000-400,000**	22(26.2)	62(73.8)	P=0.300
	**>400,000**	2(28.6)	5(71.4)	
**Creatinine**	**>132**	9(37.5)	15(62.5)	χ^2^=1.554
**(umol/L)**	**50-132**	21(26.3)	59(73.7)	P=0.460
	**<50**	1(50.0)	1(50.0)	
**CTP class**	**A**	0(0.0)	7(100.0)	χ^2^=3.332
	**B**	13(28.9)	32(71.1)	P=0.189
	**C**	18(33.3)	36(66.7)	
**Ascitic fluid protein**	**<1**	5(23.8)	16(76.2)	χ^2^=0.374
**(g/L)**	**>1**	26(30.6)	59(69.4)	P=0.541
**Upper GI Bleeding**	**YES**	6(28.6)	15(71.4)	χ^2^=0.006
	**NO**	25(29.4)	60(70.6)	P=0.940
**MELD-Na**	**<9**	0(0.0)	3(100.0)	LR=4.918
	**10-19**	6(21.4)	22(78.6)	P=0.178
	**20-29**	20(30.8)	45(69.2)	
	**30-39**	5(50.0)	5(50.0)	
	**>40**	-	-	

CTP=Child-Turcotte-Pugh; MELD-Na=Model for End-Stage Liver Disease-Sodium; INR=International Normalized ratio

**Figure 1:**
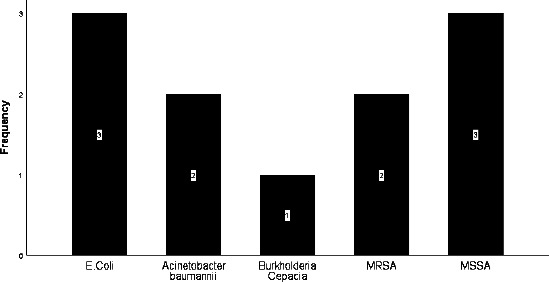
Causative organisms for SBP

Eleven study participants had positive ascitic culture results. Isolates were *Escherichia coli* 3 (27.3%), Methicillin-sensitive *Staphylococcus aureus* 3 (27.3%), Methicillin-resistant *Staphylococcus aureus* 2 (18.2%), *Acinetobacter baumanii* 2 (18.2%) and *Bulkholderia cepacia* 1 (9.1%). (Figure 1)

*Staphylococcus aureus* was the most common (5, 45.5%) isolates in the study population. Among Gram-negative bacteria isolated, the highest susceptibility was observed to Meropenem (100%), but there was absolute resistance to fluoroquinolones (ciprofloxacin, ofloxacin and levofloxacin). All the Gram-positive cocci (GPC) bacteria were susceptible to linezolid, vancomycin and daptomycin. GPC also showed high susceptibility to fluoroquinolones (80%), (Table 5).

**Table 5:** Antibiotic resistance pattern in patients with SBP (GPC: N=5, GNB: N=6)

AF antibiotic susceptibility	GPC n(%)	GNB n(%)
**Amoxicillin/Clavulanic acid**	2 (40.0)	4 (66.7)
**Gentamicin**	2 (40.0)	4 (66.7)
**Meropenem**	NT	0 (0.0)
**Ceftazidime**	NT	3 (50.0)
**Ceftriaxone**	2 (40.0)	3 (50.0)
**Ciprofloxacin**	1 (20.0)	6 (100.0)
**Ofloxacin**	1 (20.0)	6 (100.0)
**Levofloxacin**	1 (20.0)	6 (100.0)
**Linezolid**	0 (0.0)	NT
**Vancomycin**	0 (0.0)	NT
**Daptomycin**	0 (0.0)	NT

GPC=Gram positive cocci; GNB=Gram negative bacilli; NT=Not tested

*E. coli* was susceptible to meropenem (100%), and the third-generation cephalosporins (ceftriaxone and ceftazidime) (100%), but all were resistant to the fluoroquinolones (ciprofloxacin, ofloxacin, and levofloxacin) (100%). Both *A. baumannii* isolates were sensitive to meropenem, resistant to third-generation cephalosporins, fluoroquinolones, and gentamicin. Two of the five *S. aureus* strains were methicillin-resistant, showing high resistance to other tested antibiotic classes, except for linezolid, vancomycin, and daptomycin, to which they showed no resistance. The three methicillin-sensitive *S. aureus* strains were all susceptible to all the tested antibiotics (Table 6).

**Table 6:** Antibiotic-resistant pattern of bacterial isolates

Bacterial Isolates	Meropem	Ceftriaxone	Ceftazidime	Amoxicillin/Clavulinic acid	Ciprofloxacin	Ofloxacin	Levofloxacin	Gentamicin	Linezolid	Vancomycin	Daptomycin
***E. coli* (n=3)**	0(0.0)	0(0.0)	0(0.0)	2(66.7)	3(100.0)	3(100.0)	3(100.0)	1(33.3)	NT	NT	NT
***A. baumanii* (n=2)**	0(0.0)	2(100.0)	2(100.0)	1(50.0)	2(100.0)	2(100.0)	2(100.0)	2(100.0)	NT	NT	NT
***B. cepacia* (n=1)**	0(0.0)	1(100.0)	1(100.0)	1(100.0)	1(100.0)	1(100.0)	1(100.0)	1(100.0)	NT	NT	NT
**MRSA (n=2)**	NT	2(100.0)	NT	2(100.0)	1(50.0)	1(50.0)	1(50.0)	2(100.0)	0 (0.0)	0 (0.0)	0 (0.0)
**MSSA (n-3)**	NT	0(0.0)	NT	0(0.0)	0(0.0)	0(0.0)	0(0.0)	0(0.0)	0 (0.0)	0 (0.0)	0 (0.0)

*A. baumanii= Acinetobacter baumanii*, B. cepacia= *Bulkholderia cepacia*, MSSA = methicillin-sensitive *S.aureus*, MRSA= methicillin-resistant *S.aureus*; NT = Not tested (antibiotics not tested because they are not recommended for testing against the individual micro-organisms according to Clinical and Laboratory Standards Institute

## Discussion

Most of the patients studied were inpatients (66%), with the highest proportion of study participants in the age group 40-49 years, with a mean of 50.08±12.66. This finding is similar to studies in Nigeria by Ndububa *et al.*^11^ in chronic liver disease patients, Ajayi *et al.*^10^ on the prevalence of SBP in liver cirrhosis.

This age range could be due to the high level of hepatitis B infection during childhood, which accounts for most cases of liver cirrhosis and hepatocellular carcinoma in adults in this study. The majority of the study participants had liver cirrhosis in 70 (66.0%) and HCC in 36 (34%). The prevalence of SBP in this study was 29.2%, which agrees with studies done in the West that showed a prevalence ranging between 10% and 30%.^12^ However, studies done in Nigeria reported higher prevalence rates, including those by Muhammad Manko,^13^ in the north-west state of Kano, which reported a prevalence of 37.6%, and Ajayi *et al.*^10^ in Southwest, Nigeria, which reported a prevalence of 66.7%.

The report by Ajayi *et al.*^10^ was a retrospective study and also had a small sample size, which might have been responsible for the high prevalence in that study.

Most of the study patients were in CTP Class C (50.9%). This is similar to a study done by Ajayi *et al.*^10^ and also agrees with the report by Ndububa *et al.*^14^ but in contrast with a study by Muhammad Manko,^13^ which reported more patients in CTP Class B (participants were outpatients). Classical SBP accounted for 4.7%. Filik *et al.*^15^ reported a prevalence of 25.4% while Zaman *et al.*^16^ reported a prevalence of 39.28% for classical SBP in patients with suspected SBP, unlike this study, which recruited both asymptomatic and symptomatic subjects. CNNA predominates in this study (18.9%), which is similar to the study conducted by Goel et al.^17^ (60%), although at a higher percentage.

In participants with HCC, the prevalence of SBP was 27.8%, which is close to the prevalence of 20% reported by Llovet *et al.*^18^, but higher than 7.3% reported by Wang *et al.*^19^

This wide difference might be due to the number of patients with HCC, as noted by Wang *et al.*^19^ recruited 109 patients with HCC, with 8 of them having SBP.

The common clinical features of SBP in this study were generalised body weakness (28.7%), abdominal pain (32.7%), and fever (39.3%). The most common isolate in this study was *Staphylococcus aureus* 5 (45.5%), followed by E. coli 3(27.3%). *Acinetobacter baumanii* was isolated from 18.2% of positive culture reports. A study by Mittal *et al.*^20^ Regarding the microbiological profile of pathogens in spontaneous bacterial peritonitis (SBP) secondary to liver cirrhosis, Acinetobacter was also identified as one of the Gram-negative organisms, as noted by Liu *et al.*^21^ In a multicenter study on clinical characteristics of Acinetobacter bacteremia in liver cirrhosis, Acinetobacter baumanii was also reported as the causative of SBP in 33.3% of cases.

*Bulkholderia cepacia* was isolated in a patient. *Burkholderia cepacia* complex (Bcc) is a non-fermenting Gramnegative bacillus and has been rarely reported to cause spontaneous bacterial peritonitis (SBP) in decompensated cirrhosis. A study by Taneja *et al.*^22^ among 252 SBP patients, 11(4.3%) patients with a positive ascitic fluid culture for *Bulkholderia cepacia* complex. Better bacterial isolation and identification methods with BACTEC blood culture system and 24-pannel Microbact GNB24E used in this study undoubtedly helped to get the true identity of the bacterial isolates.

The percentage sensitivity pattern for Gram negative organisms was meropenem (100%), cephalosporins (50.0%), Amoxicillin/clavulinic acid (33.7%) and gentamicin (33.7%). The resistant pattern to fluoroquinlones was absolute (100%). This is similar to a study by Ghweil *et al.*^23^ in which sensitivity to meropenem was 90.9% in their study population. The study done by Muhammad Manko,^13^ in Kano, also reported the highest sensitivity pattern to cephalosporins, followed by quinolones and least with amoxicillin-clavulanate. The resistance pattern is higher than the finding in a study by Ardolino *et al.*^24^ reporting a resistance rate to quinolones of 56% in the United States of America and 27% in a study by Ghweil *et al.*^23^. All Gram-positive organisms in this study were sensitive to linezolid, vancomycin and daptomycin. Among the MSSA, sensitivity pattern was also absolute to ceftriaxone, amoxicillin-clavulanate, fluoroquinolones and gentamicin. MRSA showed high resistance to all tested antibiotics except linezolid, vancomycin and daptomycin to which they were all susceptible. This is similar to the study by Guo *et al.*^25^ conducted over 5 years involving 912 patients with liver cirrhosis and ascites in a Chinese teaching hospital reporting vancomycin and linezolid susceptibilities in all isolated Staphylococcus aureus among patients with SBP; however, the study did not report testing for daptomycin.

The difference in overall susceptibility of bacterial isolates in studies may be due to the wide variability of bacteria across studies, with differences in their intrinsic behaviour to antibiotics. In addition, high-scale and widespread use of quinolones in the prophylaxis of SBP has promoted selective pressure and flora modifications, resulting in the development of quinolone resistance, widely reported in the literature.^26^

*E. coli* has a high sensitivity to meropenem and cephalosporins which is similar to the study by Alexopoulou *et al.*^27^ and Muhammad Manko,^13^ in Kano, Nigeria. However, isolates were not tested against meropenem in the study by Muhammad Manko. *E.coli* was 100% resistant to quinolones in this study. Recent studies are now showing a changing trend in antibiotic sensitivity and resistance patterns.^28^,^29^

Multidrug–resistant organisms (MDROs) are defined, according to international guidelines, as those with acquired resistance to at least three antibiotic classes.^25^ In this study, MDR was seen with the following isolates: MRSA, *Bulkholderia cepacia, Acinetobacter baumanii*. Oliveria *et al.*^30^ reported a MDR level of 46.9%. At variance with our finding, Wen Lin Tay *et al.*^28^ found a low resistance with MRSA at 6.23% and to Vancomycin-Resistant *Enterococci* at 1.9%.

*Acinetobacter baumanii* also showed a high antitbiotic sensitivity to meropenem and moderate sensitivity to amoxicillin/clavulinic acid. A multicenter study by Chang-Pan *et al.*^21^ on the clinical characteristic of *acinetobacter* bacteremia reported a 50% sensitivity to meropenem, although the isolates were from the bloodstream, substantial level of MDR was noted to *Acinetobacter baumanii*. In literature, *Bulkholderia cepacia* has been reported as a rare cause of SBP^31^. In this study, a case of *Bulkholderia cepacia* was isolated and was sensitive to meropenem with a MDR pattern that is similar to the case report by *Hamahata et al*.^31^ however a follow-up was not done for this single case of *Bulkholderia cepacia* as this was a cross-sectional study.

Treatment guidelines recommend a third-generation cephalosporin for empiric treatment of SBP.^32^ However, the overall findings from this study's antibiotic susceptibility query the efficacy of third-generation cephalosporins as traditional empiric agents for SBP in our setting. Our experience corroborates the suggestion by a panel of experts that empiric treatment should be with broader-spectrum antibiotics, especially in patients with nosocomial SBP.

A study by Piano et al.^34^ underscores the importance of antibiotic coverage for multidrug-resistant bacteria, such as extended-spectrum beta-lactamase (ESBL)-producing Enterobacteriales, as well as methicillin-resistant Staphylococcus aureus (MRSA). This study agrees with our findings that a combination of meropenem and daptomycin has established superiority over a third-generation cephalosporin (ceftazidime).

## Conclusion

The prevalence of SBP was 29.2%. The most common variant of SBP was the CNNA subtype, accounting for 18.9% of SBP cases. The most common causative bacteria were Staphylococcus aureus, which accounted for almost half of the total isolates. GNB showed high resistance to all tested antibiotics except meropenem. Likewise, 40% of the Staphylococcus aureus were methicillin-resistant, which implies a high resistance rate to beta-lactam antibiotics and also to the tested aminoglycosides. The findings underscore the need for microbial identification and sensitivity patterns in selecting first-line antibacterial agents for treating patients with SBP in our environment. Ascitic fluid culture and antibiotic sensitivity testing should be done routinely in all patients with decompensated liver cirrhosis and HCC with ascites to inform definitive treatment.
